# Multi-Omics Analysis Reveals the IFI6 Gene as a Prognostic Indicator and Therapeutic Target in Esophageal Cancer

**DOI:** 10.3390/ijms25052691

**Published:** 2024-02-26

**Authors:** Nguyen-Kieu Viet-Nhi, Tran Minh Quan, Vu Cong Truc, Tran Anh Bich, Pham Hoang Nam, Nguyen Quoc Khanh Le, Po-Yueh Chen, Shih-Han Hung

**Affiliations:** 1 International Master/Ph.D. Program in Medicine, College of Medicine, Taipei Medical University, Taipei 110, Taiwan; d142110002@tmu.edu.tw; 2 Department of Thoracic Surgery, Cho Ray Hospital, Ho Chi Minh City 700000, Vietnam; tranminhquan.yds@gmail.com; 3 Department of Otolaryngology, Faculty of Medicine, University of Medicine and Pharmacy at Ho Chi Minh City, Ho Chi Minh City 700000, Vietnam; trucvucong@gmail.com; 4 Department of Otolaryngology, Cho Ray Hospital, Ho Chi Minh City 700000, Vietnam; trananhbich2015@gmail.com (T.A.B.); bsnamtmh@gmail.com (P.H.N.); 5 Professional Master Program in Artificial Intelligence in Medicine, College of Medicine, Taipei Medical University, Taipei 110, Taiwan; khanhlee@tmu.edu.tw; 6 AIBioMed Research Group, Taipei Medical University, Taipei 110, Taiwan; 7 Research Center for Artificial Intelligence in Medicine, Taipei Medical University, Taipei 110, Taiwan; 8 Translational Imaging Research Center, Taipei Medical University Hospital, Taipei 110, Taiwan; 9 Department of Otolaryngology, Wan Fang Hospital, Taipei Medical University, Taipei 110, Taiwan; 10 Department of Otolaryngology, School of Medicine, College of Medicine, Taipei Medical University, Taipei 110, Taiwan

**Keywords:** IFI6, esophageal cancer, prognostic indicators, gene expression, bioinformatics, multi-omics analysis

## Abstract

The role of the IFI6 gene has been described in several cancers, but its involvement in esophageal cancer (ESCA) remains unclear. This study aimed to identify novel prognostic indicators for ESCA-targeted therapy by investigating IFI6’s expression, epigenetic mechanisms, and signaling activities. We utilized public data from the Gene Expression Omnibus (GEO) and the Cancer Genome Atlas (TCGA) to analyze IFI6’s expression, clinical characteristics, gene function, pathways, and correlation with different immune cells in ESCA. The TIMER2.0 database was employed to assess the pan-cancer expression of IFI6, while UALCAN was used to examine its expression across tumor stages and histology subtypes. Additionally, the KEGG database helped identify related pathways. Our findings revealed 95 genes positively correlated and 15 genes negatively correlated with IFI6 in ESCA. IFI6 was over-expressed in ESCA and other cancers, impacting patient survival and showing higher expression in tumor tissues than normal tissues. IFI6 was also correlated with CD4+ T cells and B cell receptors (BCRs), both essential in immune response. GO Biological Process (GO BP) enrichment analysis indicated that IFI6 was primarily associated with the Type I interferon signaling pathway and the defense response to viruses. Intriguingly, KEGG pathway analysis demonstrated that IFI6 and its positively correlated genes in ESCA were mostly linked to the Cytosolic DNA-sensing pathway, which plays a crucial role in innate immunity and viral defense, and the RIG-I-like receptor (RLR) signaling pathway, which detects viral infections and activates immune responses. Pathways related to various viral infections were also identified. It is important to note that our study relied on online databases. Given that ESCA consists of two distinct subgroups (ESCC and EAC), most databases combine them into a single category. Future research should focus on evaluating IFI6 expression and its impact on each subgroup to gain more specific insights. In conclusion, inhibiting IFI6 using targeted therapy could be an effective strategy for treating ESCA considering its potential as a biomarker and correlation with immune cell factors.

## 1. Introduction

Esophageal squamous cell carcinoma (ESCC) is a malignancy that poses a significant global health burden, being both the sixth leading cause of cancer-related death worldwide and the second deadliest gastrointestinal cancer [1,2,3,4]. In cases of resectable esophageal cancer (ESCA), the primary treatment approach involves a combination of surgical intervention with or without neoadjuvant and/or adjuvant chemotherapy or chemoradiotherapy [2,5,6]. Conversely, for patients deemed inoperable, the treatment options may include chemotherapy, radiotherapy, targeted therapy, and immunotherapy [2,7,8]. Despite the availability of various treatment options, the prognosis remains bleak, with a reported five-year overall survival rate rarely exceeding 40% [2].

In recent studies, the combination of neoadjuvant chemotherapy and immune checkpoint inhibitors (ICIs) has shown significant clinical effectiveness in treating locally advanced ESCC patients. These findings have revealed a notable rate of major pathological remission (MPR) at approximately 50.0% and a pathological complete response (pCR) rate of around 30% [9,10]. Extensive evidence from neoadjuvant studies has established that the preoperative administration of immunotherapy significantly reduces the tumor size in most locally advanced ESCC patients, thereby greatly enhancing the success rate of radical surgery [10,11,12]. Nonetheless, the treatment outcomes have displayed considerable heterogeneity. In manifesting inherent resistance to immunotherapeutic interventions, the implementation of this treatment protocol may retard the progression of the patient’s pathological state, impede the feasibility of undertaking radical surgical procedures, and consequently lead to a less favorable clinical outlook. Consequently, investigating resistant biomarkers, rather than responsive biomarkers, has become a pressing clinical priority [10].

Interferon-α-inducible protein 6 (IFI6) has been extensively studied across various cancer types, such as lung adenocarcinoma, myeloma, gastric cancer, pancreatic cancer, breast cancer, prostate cancer, and ovarian cancer [13,14,15,16,17,18,19,20]. Currently, the amount of research exploring the correlation between IFI6 and ESCA is insufficient, both in in vivo and in vitro settings. However, a few recent studies have brought attention to the involvement of IFI6 as a combined biomarker within a specific subgroup of patients with ESCC. These investigations have uncovered that IFI6 participates in immunotherapeutic resistance and unfavorable survival rates by adjusting the mesenchymal and immune-suppressive microenvironment according to molecular-specific mechanisms [10,21].

The contribution of the IFI6 gene in ESCA remains unclear. In this study, to unravel its potential role, we conducted a comprehensive multi-omics bioinformatics analysis dedicated to investigating the involvement of the IFI6 gene in ESCA. By utilizing publicly available data from the Gene Expression Omnibus (GEO) and the Cancer Genome Atlas (TCGA), we examined IFI6’s expression level, epigenetic mechanisms, and signaling activities within ESCA. Furthermore, we explored correlations between IFI6 and various immune cells while also assessing its pan-cancer expression using multiple tools.

## 2. Results

### 2.1. Screening of IFI6 Gene and Protein Information

Brief gene and protein information on IFI6 is shown in Table 1.

IFI6, also referred to as G1P3, ISG16, or IFI-6-16, belongs to the FAM14 family of interferon-stimulated genes (ISGs), which includes four genes in humans (IFI6, IFI27, IFI27L1, and IFI27L2) [22,23]. It is a hydrophobic protein of a 13 kDa size, consisting of 130 amino acids and containing putative transmembrane helices [23,24]. IFI6 is primarily localized in the inner mitochondrial membrane.

**Figure 1:**
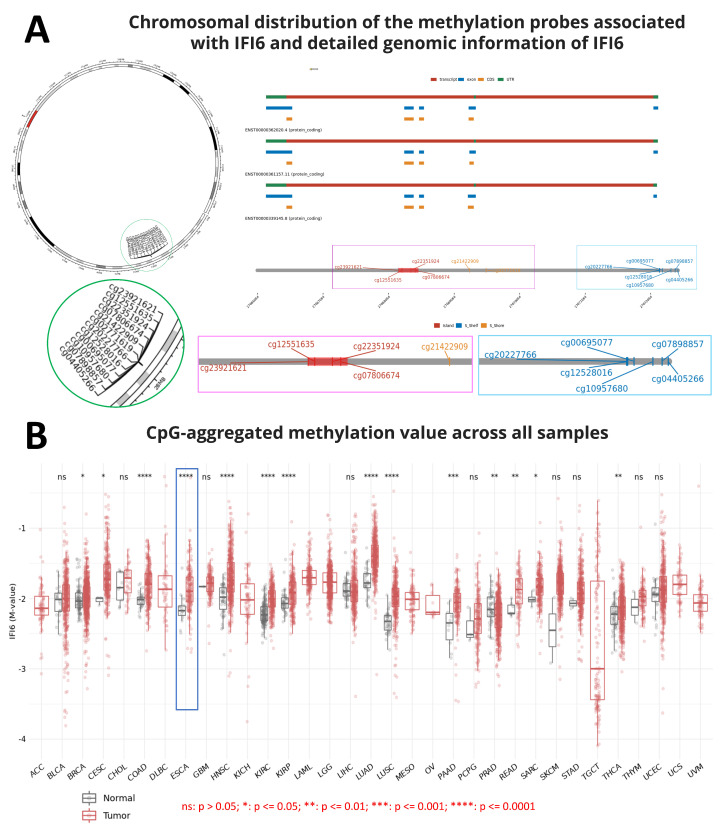
(**A**) Chromosomal distribution of the 12 methylation probes associated with IFI6 and detailed genomic information of IFI6. (**B**) CpG-aggregated methylation value across different tumor samples (*n* = 9602 samples total). Methylation associated with IFI6 in ESCA shows significant differences between tumor and normal samples (ns: *p* > 0.05; *: *p* ≤ 0.05; **: *p* ≤ 0.01; ***: *p* ≤ 0.001; ****: *p* ≤ 0.0001).

Nowadays, the role of methylation in gene expression has been reported in several studies. Methylation was believed to play a crucial role in repressing gene expression and cell differentiation. DNA methylation governs gene expression by recruiting proteins associated with gene repression or by impeding the binding of transcription factor(s) to DNA [25]. Indeed, errors in methylation might lead to various diseases, such as cancer [25,26,27]. Hence, during the process of screening the IFI6 gene information, we aimed to gain an overview of the chromosomal distribution of the methylation probes associated with IFI6 and examine the aggregated CpG methylation values in ESCA and other cancers. Figure 1A illustrates the chromosomal distribution of 12 methylation probes linked to IFI6. These probes, namely cg23921621, cg12551635, cg22351924, cg07806674, cg20227766, cg12528016, cg00695077, cg10957680, cg07898857, cg04405266, cg21422909, and cg02771619, are located on Chromosome 1.

Additionally, our analysis revealed that the CpG-aggregated methylation value of IFI6 in ESCA tumors is significantly higher compared to in normal samples (Figure 1B). Among a total of 9602 normal and tumor samples analyzed across different cancers, significant differences were observed in the CpG-aggregated methylation values associated with IFI6 in ESCA between the tumor and normal samples (**** *p* ≤ 0.0001) (Supplementary Materials file). CpG islands, situated in the promoter regions, exert regulatory control over gene expression by suppressing the associated gene [28]. Consequently, DNA methylation at the CpG islands emerges as a crucial determinant of gene expression and tissue-specific physiological processes [28].

### 2.2. IFI6 Is an Over-Expressed Gene in ESCA

**Figure 2:**
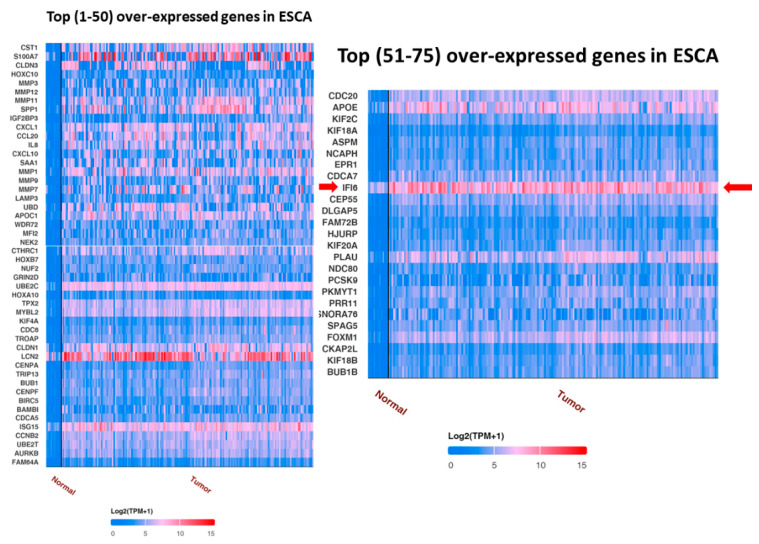
Top 75 over-expressed genes in ESCA. IFI6 is an over-expressed gene in esophageal carcinoma (red arrow).

UALCAN analysis showed the top over-expressed and under-expressed genes in ESCA compared to in normal samples. IFI6 is an over-expressed gene in ESCA (Figure 2).

**Figure 3:**
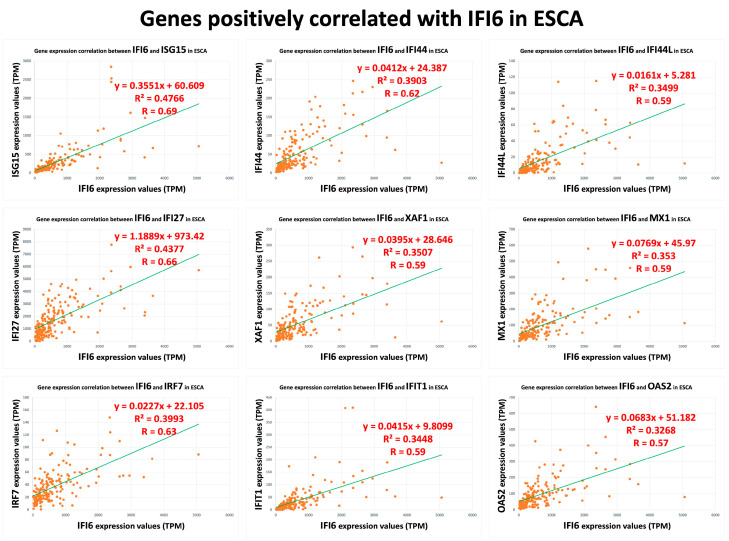
Top nine positively correlated genes with IFI6 in ESCA.

In our analysis, we identified a list of 95 genes that showed a positive correlation with the IFI6 gene in ESCA. Among these genes, we selected the top nine based on their strength of correlation as measured using Pearson’s correlation coefficient (R). The plots of these top nine positively correlated genes (ISG15, IFI27, IRF7, IFI44, XAF1, IFIT1, IFI44L, MX1, OAS2) are presented in Figure 3. These found genes, ISG15 (ISG15 ubiquitin-like modifier), IFI27 (interferon alpha-inducible protein 27), IFI44L (interferon-induced protein 44-like), IFI44 (interferon-induced protein 44), IFIT1 (interferon-induced protein with tetratricopeptide repeats 1), MX1 (myxovirus (influenza virus) resistance 1), and IFI6, are used to determine the IFN-I signature [29,30,31]. Interestingly, they are also the common interferon-α-inducible genes in systemic lupus erythematosus patients [29,30,31].

**Figure 4:**
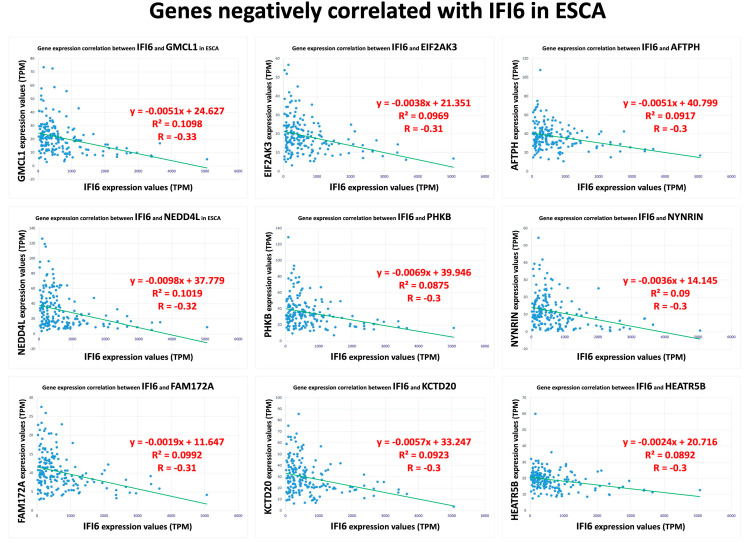
Top nine negatively correlated genes with IFI6 in ESCA.

Furthermore, we also identified a separate set of genes that exhibited a negative correlation with the IFI6 gene in ESCA. The top nine negatively correlated genes (GMCL1, NEDD4L, FAM172A, EIF2AK3, PHKB, KCTD20, AFTPH, NYNRIN, HEATR5B) are displayed in Figure 4.

### 2.3. High Expression Levels of IFI6 in ESCA Show the Association with Poor Clinical Outcomes, including Advanced Disease Stage and Increased Risk of Metastasis

**Figure 5:**
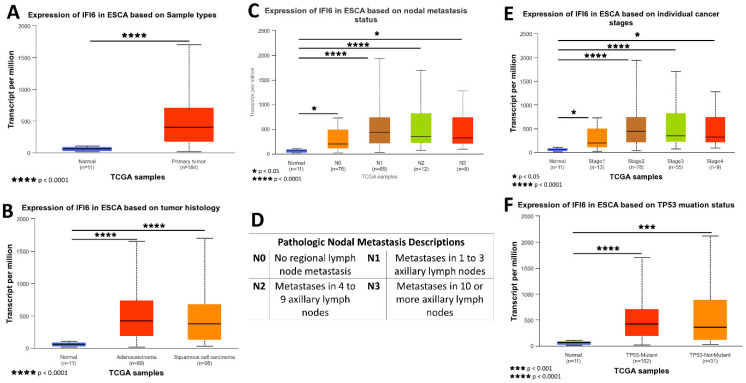
Comprehensive insights into the expression of IFI6 gene in ESCA based on sample types (**A**), tumor histology (**B**), nodal metastasis status (**C**), individual cancer stages (**E**), and TP53 mutation status (**F**); pathologic nodal metastasis description table (**D**).

The gene expression data and clinical information about patients were acquired from TCGA and subjected to analysis using the UALCAN platform. A box plot comparing the relative expression of IFI6 in normal and ESCA samples is depicted in Figure 5A. Notably, the expression of IFI6 demonstrates a statistically significant difference between normal samples and both EAC and ESCC (Figure 5B), based on tumor histology.

To assess the impact of IFI6 expression on different cancer stages, AJCC (the American Joint Committee on Cancer) pathologic tumor stage information was utilized. The samples were categorized into four groups: stage I, stage II, stage III, and stage IV (Figure 5E). The results indicate that high expression levels of IFI6 in ESCA are associated with poorer tumor stages and an increased risk of nodal metastasis (Figure 5C).

Additionally, the analysis reveals a higher expression of IFI6 in both TP53 mutant and non-TP53-mutant samples in ESCA, indicating a potential association between IFI6 expression and TP53 status (Figure 5F).

These findings suggest that elevated levels of IFI6 expression in ESCA might indicate a poor tumor stage and increased nodal metastasis risk and potentially correlate with TP53 mutation status.

### 2.4. The Expression of IFI6 Is Associated with the Overall Survival of ESCA Patients

ESCA is a prevalent form of cancer that is associated with a bleak prognosis [32]. Among the subtypes of ESCA, ESCC is primarily associated with risk factors such as tobacco smoking, alcohol consumption, hot beverage drinking, and poor nutrition [32,33]. Conversely, EAC primarily affects individuals of white ethnicity, and the risk factors include smoking, obesity, and gastroesophageal reflux disease [33,34].

**Figure 6:**
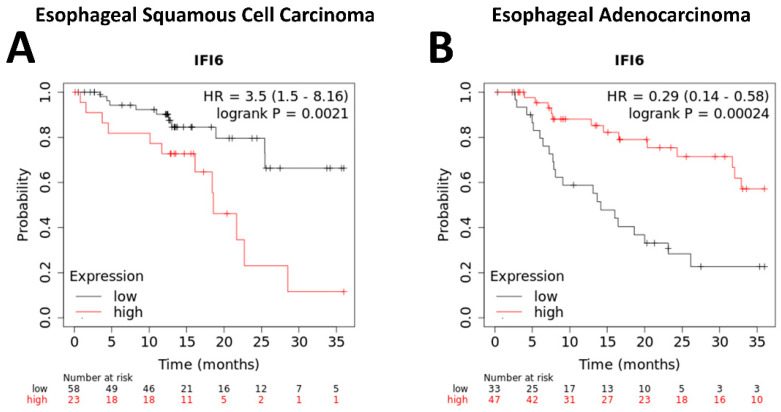
Kaplan–Meier plotter analysis of overall survival of ESCC (**A**) and EAC (**B**) patients based on IFI6 gene expression levels.

Our study indicated that higher expression levels of IFI6 have been observed to correlate with poorer overall survival rates in ESCC patients (Figure 6A). This suggests that increased IFI6 expression might indicate a higher risk of adverse outcomes and reduced survival in ESCC. However, the results in EAC show the opposite outcome (Figure 6B).

### 2.5. IFI6 Expression Correlates with Immune Infiltrates in ESCA

**Figure 7:**
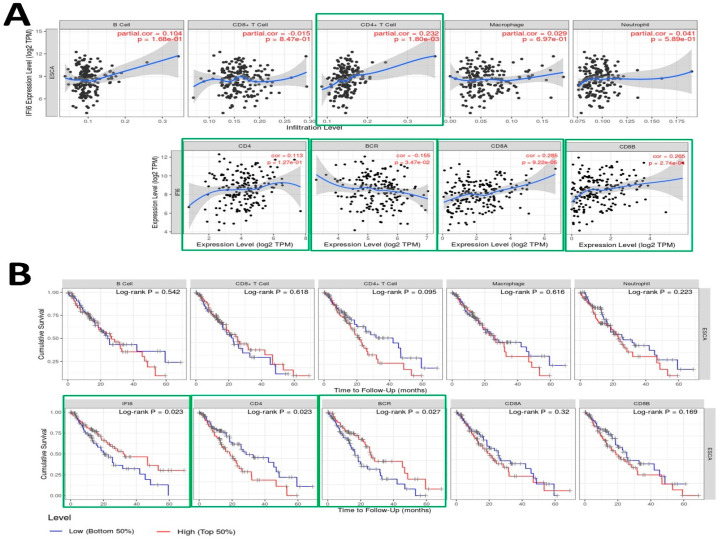
(**A**) Examination of the correlation between IFI6 gene expression and various immune infiltrates in esophageal carcinoma (ESCA), including B cells (*p* = 0.168), CD8+ T cells (*p =* 0.847), CD4+ T cells (*p* = 0.0018), Macrophages (*p* = 0.697), Neutrophils (*p* = 0.589), CD4 (*p* = 0.127), B cell receptors (BCRs) (*p* = 0.0347), CD8A (*p* = 0.0000922), and CD8B (*p* = 0.000274). (**B**) Utilization of Kaplan–Meier plots for immune infiltrates and genes to illustrate survival differences in ESCA, incorporating the log-rank test-derived *p*-value.

Employing the TIMER tool, we executed an analysis to investigate the relationship between the expression of the IFI6 gene and diverse immune infiltrates in esophageal carcinoma (ESCA), encompassing B cells, CD4+ T cells, CD8+ T cells, neutrophils, macrophages, B cell receptors (BCR), CD4, CD8A, and CD8B (Figure 7A).

The analysis revealed significant correlations between IFI6 expression and immune response in ESCA. Specifically, IFI6 showed a significant correlation with CD4+ T cells (*p* = 0.0018) and B cell receptors (BCRs) (*p* = 0.0347), both of which play crucial roles in the immune response. CD4+ T cells are important for coordinating immune responses [35], while B cells recognize antigens through their B cell receptors (BCRs) and are activated to produce antibodies [36]. IFI6 also showed a significant correlation with CD8A and CD8B cells, which are two closely linked genes in the CD8 locus and serve as co-receptors for T cell receptors (TCRs) [37,38]. Therefore, elevated IFI6 expression could potentially impact the functionality of both B cell receptors (BCRs) and T cell receptors (TCRs), influencing immune surveillance, tumor infiltration, and the overall immune response dynamics. These observations suggest that IFI6 might be involved in modulating immune responses within the tumor microenvironment of ESCA.

In addition to analyzing the correlation between the immune gene expression levels and cumulative survival in ESCA, we conducted univariate Cox regression analysis to investigate their prognostic significance further. Table 2 presents the results, including each immune gene’s coefficient, HR, 95% CI, and *p*-value. To visually depict the survival differences, we generated Kaplan–Meier plots for the immune infiltrates and genes (Figure 7B). The plots were divided into two groups based on user-defined cutoffs to classify the expression levels as low and high. The *p*-value obtained using the log-rank test, which compares the survival curves of the two groups, is displayed on each plot. Remarkably, our findings indicate that in addition to IFI6, the CD4 and BCR expression levels also exhibit a significant correlation with the cumulative survival in ESCA.

### 2.6. Pathway Analysis Reveals Common Pathways Associated with IFI6 and Its Positively Correlated Genes

**Figure 8:**
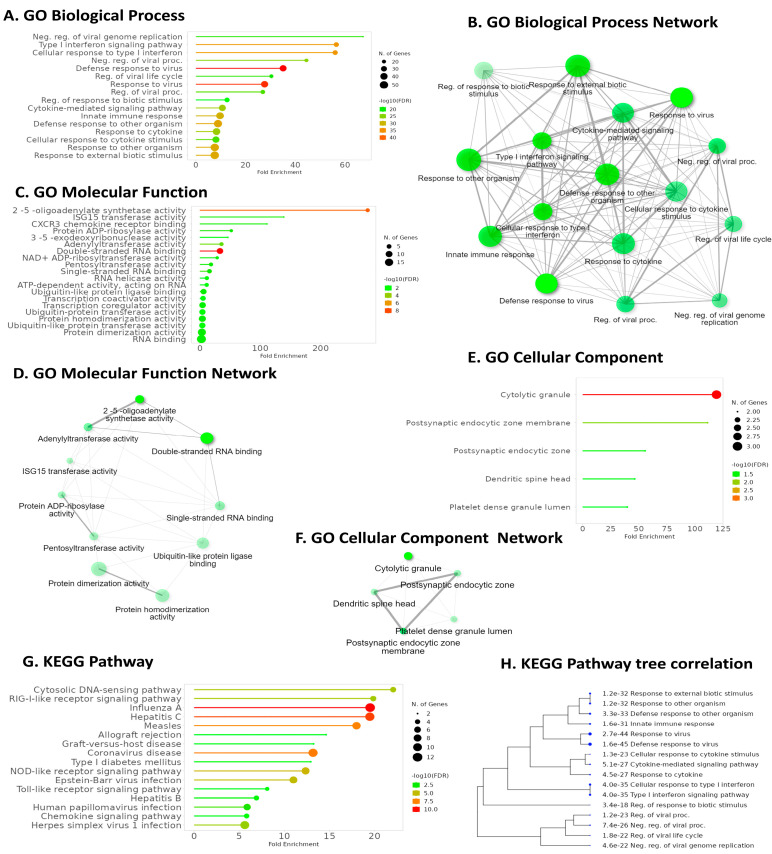
Key pathways were identified using GO and KEGG enrichment analysis for IFI6 and its positively correlated genes. (**A**) Enriched GO biological processes. (**B**) Network visualization of interconnected pathway networks in GO Biological Process; the nodes represent pathways and interconnections depict pathway associations. (**C**) Enriched GO molecular functions. (**D**) Network visualization of interconnected pathway networks in GO Molecular Functions. (**E**) Enriched GO cellular components and their networks (**F**). (**G**) KEGG pathway enrichment analysis. (**H**) Hierarchical clustering tree summarizing correlation among significant pathways in KEGG enrichment analysis. Pathways with evaluated genes are clustered together, and the larger dots indicate more significant *p*-values.

GO Biological Process (GO BP) enrichment analysis highlighted IFI6’s association with the defense response to viruses, emphasizing its role in antiviral immunity (Figure 8A). GO Molecular Function (GO MF) analysis revealed a significant pathway related to double-stranded RNA binding, further elucidating IFI6’s involvement in recognizing viral genetic material (Figure 8C). Intriguingly, KEGG pathway analysis demonstrated that IFI6 and its positively correlated genes in ESCA were predominantly associated with the Cytosolic DNA-sensing pathway, a key player in innate immunity and viral defense (Figure 8G). Additionally, the RIG-I-like receptor (RLR) signaling pathway, responsible for detecting viral infections and activating immune responses, strongly correlated with IFI6. Furthermore, the analysis identified pathways linked to various viral infections, including Hepatitis C, Influenza A, Coronavirus, Human Papillomavirus, and Herpes simplex virus I infection.

## 3. Discussion

In this investigation, we analyzed IFI6’s expression, epigenetic mechanisms, and signaling activities using multi-omics techniques. Our findings substantiate that targeted therapy aimed at inhibiting IFI6 may prove to be an efficacious approach to addressing ESCA owing to its potential as a biomarker and its correlation with immune cell factors.

**Figure 9:**
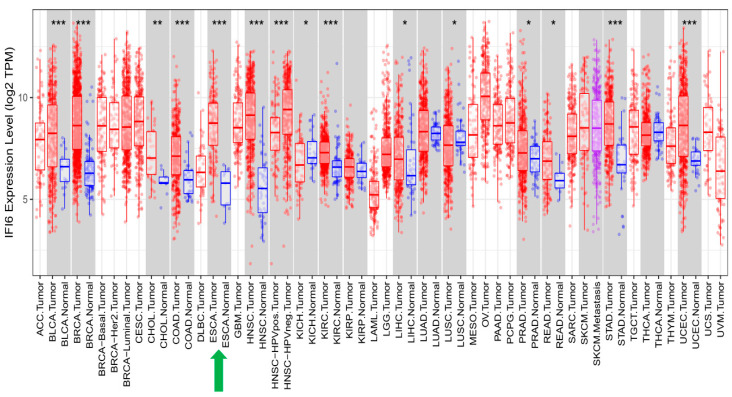
In addition to ESCA, IFI6 expression levels are significantly elevated in various other cancer types when compared to normal samples (*: *p* ≤ 0.05; **: *p* ≤ 0.01; ***: *p* ≤ 0.001).

IFI6 has been reported to play an essential role in different cancers [13,14,15,16,17,18,19,20]. Similarly, we have shown the highly significant impact of high IFI6 expression levels on various types of cancer (Figure 9). In addition to ESCC, these other cancers include Bladder Cancer (BLCA), Breast Cancer (BRCA), Cholangiocarcinoma (CHOL), Colon Cancer (COAD), Head and Neck Cancer (HNSC), Kidney Renal Clear Cell Carcinoma (KIRC), Stomach Adenocarcinoma (STAD), and Uterine Corpus Endometrial Carcinoma (UCEC).

Our research revealed that the upregulation of IFI6, with the alias G1P3, a member of the FAM14 family of interferon-stimulated genes (ISGs), was conspicuously observed in ESCA patients. Furthermore, this heightened expression was predominantly localized within the inner mitochondrial membrane. Recent studies have demonstrated the role of mitochondrial reactive oxygen species (mtROS) in metastasis. Due to IFI6 being located in the mitochondrial membrane and its elevated expression being associated with poor prognosis, numerous research works have been conducted to demonstrate the relationship between IFI6 and mitochondrial reactive oxygen species (mtROS) in cancer treatment mechanisms: Venugopalan Cheriyath et al. reported that G1P3 (IFI6)-induced mitochondrial reactive oxygen species (mtROS) play a direct role in the formation of migratory structures and the expression of nuclear genes, promoting metastasis in breast cancer cells [14]. Additionally, they suggested that targeting the mitochondrial functions of G1P3 may potentially improve the clinical outcomes in breast cancer patients [14]. In a study by Chu Lv and Meng Li, the effect of IFI6 in colorectal cancer (CRC) was analyzed [16]. They found that knockdown of IFI6 inhibited the cell viability and colony formation while inducing cell apoptosis through the upregulation of the p53/p21 signaling pathway [16]. Zhenchuan Liu et al. investigated the mechanism of ESCC and its modulation according to redox signals [21]. Their findings revealed that IFI6 contributed to ESCC cell proliferation and survival by modulating redox homeostasis [21]. The downregulation of IFI6 resulted in mitochondrial calcium overload mediated by the mitochondrial calcium uniporter (MCU), inhibited mitochondrial supercomplex assembly, and suppressed the respiratory phosphorylation efficiency, ultimately leading to elevated mitochondrial ROS production [21]. However, Zhenchuan Liu et al. only focused on the study of ESCC, which is one subtype of ESCA [21]. It is crucial to conduct more comprehensive studies encompassing the remaining subtypes in the future.

Our study demonstrates the correlation between IFI6 and immune genes such as CD4+, BCR, CD8A, and CD8B genes in ESCA. CD4+ T cells and CD8+ T cells constitute the majority of T lymphocytes [39]. B cell receptors (BCRs) are transmembrane proteins found on the surface of B cells [40]. Our research has revealed that IFI6 interacts with both the humoral and cell-mediated immune responses in ESCC. T cells predominantly identify intracellular pathogens and abnormal cells, thereby contributing to cellular immunity. In contrast, B cells possess the capacity to recognize a diverse range of antigens and are pivotal in generating antibodies as part of the humoral immune response. These two cell types work in synchrony to provide a comprehensive immune response to pathogens [39,40]. Laura Villamayor et al. have reported that IFI6 plays a role as a negative regulator of innate immune responses by modulating the activation of retinoic acid-inducible gene I (RIG-I) [23]. RIG-I is a crucial sensor for viral infections, and it orchestrates the transcription of interferons (IFNs) and inflammatory proteins. However, an excessive immune response is not good [23]. By knocking down the expression of IFI6, they observed a reduction in the production of infectious Influenza A Virus (IAV) and SARS-CoV-2. This newly discovered function of IFI6 holds promise as a therapeutic target for addressing conditions marked by overactive innate immune responses and combating viral infections [23].

An intriguing discovery in our investigation lies in the linkage uncovered using KEGG analysis between IFI6 and its genes that positively correlate with the Hepatitis C pathway. HCV is widely recognized as a primary contributor to mortality resulting from liver-related complications across the globe. Nevertheless, various recent studies conducted in Denmark, Canada, and the USA involving large population-based case–control and cohort investigations have observed a relationship between infection with HCV and an elevated frequency of extrahepatic cancer such as pancreatic cancer, kidney cancer, lung cancer, and oropharyngeal cancer [41,42,43,44]. This raises the question of whether Hepatitis C is also associated with ESCA. Indeed, recent reports have indicated a modest association between chronic HCV infection and the development of ESCA. Additionally, studies suggest that interferon-based anti-HCV therapy may reduce the risk of ESCA and the overall mortality in individuals infected with HCV [44,45]. These findings underscore the need for further research to explore the relationship between Hepatitis C and ESCA in more detail.

There are certain limitations to the study that merit discussion. Firstly, the analysis relies heavily on publicly available datasets from GEO, TCGA, and other databases, which may have inherent biases or inaccuracies. The retrospective nature of these datasets may not capture the dynamic interactions of IFI6’s gene expression, epigenetic modifications, and immune responses over time. Moreover, the utilization of bioinformatics platforms for data analysis, although powerful, can sometimes oversimplify complex biological interactions. The correlation findings between IFI6, immune cells, and the associated pathways are associative and not causative. Hence, the mechanisms underlying these correlations require further validation using experimental studies. Additionally, the study does not provide in vivo or in vitro evidence supporting the clinical relevance of IFI6, and the findings are not validated in an independent cohort, which is crucial for establishing the robustness and reproducibility of the results. The study also does not delve into the potential off-target effects of inhibiting IFI6, which could be a significant consideration for its therapeutic targeting. The discrepancies in the prognostic significance of IFI6 expression between ESCA subtypes also warrant a more in-depth investigation to understand the underlying heterogeneity. Lastly, the study does not account for other possible confounding factors, such as different treatment regimens, genetic backgrounds, and environmental factors that might influence IFI6 expression and its association with clinical outcomes in ESCA. These limitations underscore the need for further comprehensive research, including prospective studies and experimental validation, to ascertain the clinical utility of IFI6 as a therapeutic target or biomarker in ESCA.

It should be noted that this study relies on data primarily obtained from online databases. As ESCA can be classified into two distinct subgroups, ESCC and EAC, and most of the online databases we used combine these two subgroups into a single disease category, it is crucial for future research to evaluate the impact of IFI6 expression on each individual cancer subgroup to gain more understanding.

Despite its limitations, our study provides a comprehensive understanding of the pivotal role played by the IFI6 gene in ESCA. It delves into the over-expression of IFI6 in this disease and its consequential impact on disease progression, metastasis, and overall survival. These findings set the stage for researchers to further hypothesize and conduct in vitro and in vivo experiments that focus on modulating mitochondrial function in ESCA via IFI6 gene manipulation and potential targeted therapies involving immune cells and associated pathways. Evidently, IFI6 emerges as a promising biomarker for predicting and treating ESCA.

## 4. Materials and Methods

### 4.1. Gene and Protein Information

The Human Protein Reference Database (HPRD) (http://www.hprd.org, accessed on 16 July 2023) is a valuable web-based resource that provides comprehensive information on various aspects of human proteins [46]. In this study, HPRD was used to gather relevant gene and protein information specific to IFI6.

The DNA methylation phenotype of IFI6 in ESCA was investigated based on SMART (Shiny Methylation Analysis Resource Tool) (http://www.bioinfo-zs.com/smartapp, accessed on 16 July 2023) and TCGA data [27,47].

### 4.2. UALCAN Analysis

UALCAN was used to access to the publicly available cancer OMICS data (TCGA) and reveal the expression of IFI6 across different cancer types [48,49].

Firstly, we accessed the UALCAN analysis page and selected ESCA as our target cancer type. Subsequently, we evaluated the expression status of our gene of interest, IFI6, to determine whether it is over-expressed or under-expressed in ESCA. Additionally, we utilized UALCAN to identify the top genes that showed a positive or negative correlation with IFI6 in ESCA. Among the top genes that exhibited a positive correlation with IFI6, we further explored the pathways associated between them in the context of ESCA.

To gain more comprehensive insights into the expression of IFI6 in ESCA, we assessed its expression based on various factors, including sample types, individual cancer stages, tumor grade, histology type, nodal metastasis status, and TP53 mutation status.

### 4.3. Survival Analysis

Kaplan-Meier plotter is a valuable tool for discovering and validating survival biomarkers by assessing the correlation between gene expression and patient survival across various tumor types [50]. To conduct survival analysis for ESCA, we utilized the Kaplan-Meier plotter tool (https://kmplot.com/analysis/, accessed on 16 July 2023) and entered “IFI6” as the gene of interest for analysis.

ESCA encompasses two primary histological subcategories: ESCC and esophageal adenocarcinoma (EAC). These subtypes exhibit different clinicopathological characteristics and unique features. In this analysis, we focused on evaluating the influence of IFI6 gene expression on patient groups with ESCC and EAC.

Using the Kaplan-Meier plotter tool, we obtained survival curves that provided insights into the correlation between IFI6 gene expression and the survival outcomes of ESCC and EAC patients. These curves helped assess the prognostic significance of IFI6 in each subtype of ESCA, shedding light on its potential role as a survival biomarker.

### 4.4. TIMER Analysis

The TIMER algorithm (https://cistrome.shinyapps.io/timer/, accessed on 16 July 2023) is a valuable resource that provides information on the abundance of six immune infiltrates in various cancer types. We utilized TIMER to investigate the correlation between the expression of the IFI6 gene and other crucial immune infiltrates, including B cells, CD4+ T cells, CD8+ T cells, neutrophils, macrophages, B cell receptors (BCRs), CD8A cells, and CD8B cells [27]. Using scatterplots, we visualized these correlations, allowing for a better understanding of the immune landscape in ESCA.

Additionally, we analyzed the distribution of IFI6 expression levels across multiple TCGA tumors using box plots. Within this analysis, we were able to assess the statistical significance of differential expression by employing the Wilcoxon test. By studying the expression patterns of IFI6, we can gain valuable insights into its potential role and significance in a wide range of cancer types.

### 4.5. GO and KEGG Functional Enrichment Analysis

In our study, we utilized ShinyGO version 0.77, a valuable and accessible tool in the field of bioinformatics and genomics, available at http://bioinformatics.sdstate.edu/go/, accessed on 16 July 2023. This tool integrates the Gene Ontology (GO) and Kyoto Encyclopedia of Genes and Genomes (KEGG) resources to provide comprehensive functional annotations and pathway information.

First, we employed GO to acquire structured and standardized annotations for the IFI6 gene and its positively correlated genes in ESCA. The IFI6 gene and its associated genes were categorized into two main domains: Biological Process and Molecular Function.

Additionally, we utilized KEGG, a knowledge base that focuses on understanding biological systems at the molecular level [51]. KEGG offers a collection of pathway maps that illustrate various cellular processes, signaling pathways, and metabolic pathways. In our study, we explored the pathway maps associated with the IFI6 gene and its positively correlated genes in ESCA, as provided by the KEGG database.

### 4.6. Statistical Analysis

In the survival analysis, the association between a particular variable and the overall survival outcomes was investigated according to computation of the hazard ratio (HR) along with a 95% confidence interval (CI) and log-rank *p*-value. Gene pairs with Pearson’s correlation coefficients equal to or exceeding 0.3 were deemed positive correlations, while those with coefficients of −0.3 or lower were regarded as negative correlations [49].

To investigate the differences in means between two groups, both a t-test and the Wilcoxon test were conducted. T-tests assume normality and compare the means of two groups, while the Wilcoxon test is a non-parametric test that assesses the differences in medians. For both tests, the *p*-values were computed to determine the statistical significance of the observed differences.

To indicate the significance levels in our results, we used the following convention for *p*-value reporting: **** *p* < 0.0001; *** *p* < 0.001; ** *p* < 0.01; * *p* < 0.05.

## 5. Conclusions

**Figure 10:**
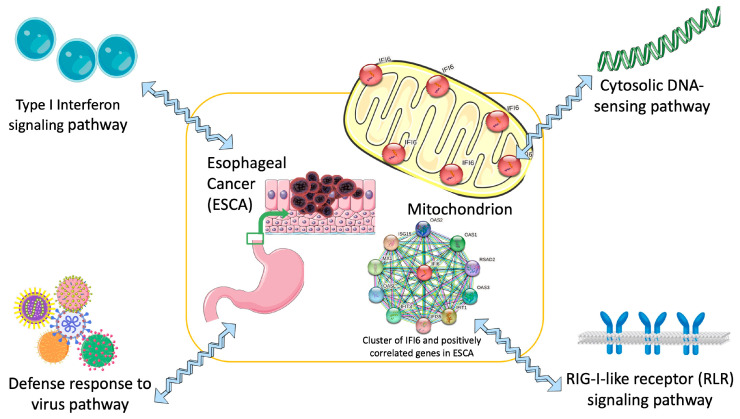
Concise diagram illustrating IFI6 as a targeted therapy for the treatment of ESCA (esophageal cancer) based on identified key signaling pathways.

Our research offers a groundbreaking perspective on the significance of the IFI6 gene in ESCA, highlighting its potential as both a prognostic biomarker and a therapeutic target. The outcomes of our study have the potential to catalyze innovative strategies for managing ESCA (Figure 10).

## Figures and Tables

**Table 1:** IFI6 gene and protein information.

**General**			
HPRD ID:	00959	Molecular Weight (Da):	13,298
Gene Symbol:	IFI6	Gene Map Locus:	1p35
Molecular Class:	Unclassified	Molecular Function:	Cytokine activity
Biological Process:	Anti-apoptosis; Immune response		
**Localization**			
Primary	Mitochondrion		
**Domains and Motifs**			
Domains		Motifs	
Transmembrane Domain (TM)	48–70	Signal Peptide (SP)	1–23
**Alternate Names**			
Alternate Names		G1P3; ISG16	
		Interferon alpha inducible protein	
		Interferon induced protein IFI-6–16	
Isoform Specific Alternate Names		Interferon induced 6–16 protein isoform b	

**Table 2:** Univariate Cox regression analysis of interested immune genes with overall survival in ESCA based on the TCGA dataset.

**Gene**	**Coefficient**	**Hazard Ratio**	**95% Confidence Interval**	***p*-Value**
B cell	−7.073	0.001	0.000–1.500300 × 10^1^	0.156
CD8+ T cell	1.648	5.196	0.000–2.314727 × 10^5^	0.763
CD4+ T cell	−2.250	0.105	0.000–1.197976 × 10^6^	0.786
Macrophage	−13.143	0.000	0.000–2.780000 × 10^−1^	0.030 *
Neutrophil	−3.016	0.049	0.000–2.038430 × 10^8^	0.790
IFI6	−0.128	0.880	0.759–1.019000 × 10^0^	0.088
CD4	0.617	1.853	1.211–2.834000 × 10^0^	0.004 **
BCR	−0.760	0.468	0.310–7.050000 × 10^−1^	0.000 ***
CD8A	0.052	1.054	0.699–1.589000 × 10^0^	0.803
CD8B	−0.091	0.913	0.573–1.454000 × 10^0^	0.702

Rsquare = 0.111 (max possible = 9.73 × 10^−1^), Likelihood ratio test *p* = 1.66 × 10^−2^, Wald test *p* = 2.58 × 10^−2^. Score (log-rank) test *p* = 1.88 × 10^−2^. (*: *p* ≤ 0.05; **: *p* ≤ 0.01; ***: *p* ≤ 0.001).

## Data Availability

The original contributions presented in the study are included in the article/Supplementary Material, further inquiries can be directed to the corresponding authors.

## Supplementary Materials

The supporting information can be downloaded at https://www.mdpi.com/article/10.3390/ijms25052691/s1.
